# Increasing importance of anthelmintic resistance in European livestock: creation and meta-analysis of an open database

**DOI:** 10.1051/parasite/2020062

**Published:** 2020-12-04

**Authors:** Hannah Rose Vineer, Eric R. Morgan, Hubertus Hertzberg, David J. Bartley, Antonio Bosco, Johannes Charlier, Christophe Chartier, Edwin Claerebout, Theo de Waal, Guy Hendrickx, Barbara Hinney, Johan Höglund, Jožica Ježek, Martin Kašný, Orla M. Keane, María Martínez-Valladares, Teresa Letra Mateus, Jennifer McIntyre, Marcin Mickiewicz, Ana Maria Munoz, Clare Joan Phythian, Harm W. Ploeger, Aleksandra Vergles Rataj, Philip J. Skuce, Stanislav Simin, Smaragda Sotiraki, Marina Spinu, Snorre Stuen, Stig Milan Thamsborg, Jaroslav Vadlejch, Marian Varady, Georg von Samson-Himmelstjerna, Laura Rinaldi

**Affiliations:** 1 Department of Infection Biology and Microbiomes, Institute of Infection, Veterinary and Ecological Sciences, University of Liverpool Neston, Cheshire CH64 7TE UK; 2 Institute for Global Food Security, Queen’s University Belfast, Biological Sciences 19 Chlorine Gardens Belfast BT9 5DL UK; 3 Institute of Parasitology, University of Zurich 8057 Zurich Switzerland; 4 Disease Control, Moredun Research Institute, Pentlands Science Park, Bush Loan Penicuik, Edinburgh EH26 0PZ UK; 5 University of Naples Federico II, Unit of Parasitology and Parasitic Diseases, Department of Veterinary Medicine and Animal Production, CREMOPAR Via Delpino, 1 80137 Napoli Italy; 6 Kreavet Hendrik Mertensstraat 17 9150 Kruibeke Belgium; 7 BIOEPAR, INRAE, Oniris 44307 Nantes France; 8 Laboratory for Parasitology, Faculty of Veterinary Medicine, Ghent University B9820 Merelbeke Belgium; 9 School of Veterinary Medicine, University College Dublin Dublin D04 W6F6 Ireland; 10 Avia-GIS Risschotlei 33 2980 Zoersel Belgium; 11 Institute of Parasitology, Department of Pathobiology, Vetmeduni Vienna Veterinärplatz 1 1210 Vienna Austria; 12 Swedish University of Agricultural Sciences, Department of Veterinary Public Health, Section for Parasitology P.O. Box 7036 Uppsala Sweden; 13 Clinic for Reproduction and Large Animals, Veterinary faculty, University of Ljubljana Gerbičeva 60 1000 Ljubljana Slovenia; 14 Department of Botany and Zoology, Faculty of Science, Masaryk University Brno 611 37 Czech Republic; 15 Animal Bioscience Department, Teagasc Grange, Dunsany, Co. Meath C15 PW93 Ireland; 16 Instituto de Ganaderia de Montana, CSIC Universidad de León 24346 Grulleros León Spain; 17 CISAS – Centre for Research and Development in Agrifood Systems and Sustainability, Escola Superior Agrária, Instituto Politécnico de Viana do Castelo, Rua Escola Industrial e Comercial de Nun’Àlvares 4900-347 Viana do Castelo Portugal; 18 EpiUnit – Instituto de Saúde Pública da Universidade do Porto Rua das Taipas, nº 135 4050-091 Porto Portugal; 19 Institute of Biodiversity, Animal Health and Comparative Medicine, University of Glasgow, Garscube Estate Glasgow G61 1QH UK; 20 Division of Veterinary Epidemiology and Economics, Institute of Veterinary Medicine, Warsaw University of Life Sciences Nowoursynowska 159c 02-776 Warsaw Poland; 21 Faculdade de Medicina Veterinária – Universidade Lusófona de Humanidades e Tecnologias Av. Campo Grande 376 1749-024 Lisbon Portugal; 22 Institute for Production Animal Clinical Science, Faculty of Veterinary Medicine, Norwegian University of Life Sciences Sandnes 4325 Norway; 23 Department of Biomolecular Health Sciences, Division Infectious Diseases and Immunology, Faculty of Veterinary Medicine, Utrecht University Yalelaan 1 3584 CL Utrecht The Netherlands; 24 Institute for Microbiology and Parasitology, Veterinary Faculty, University of Ljubljana Gerbičeva 60 1000 Ljubljana Slovenia; 25 Department of Veterinary Medicine, Faculty of Agriculture, University of Novi Sad 21101 Novi Sad Republic of Serbia; 26 Veterinary Research Institute, Section for Parasitology, HAO-DEMETER, Thermi 57001 Thessaloniki Greece; 27 Department of Clinical Sciences, Faculty of Veterinary Medicine, University of Agricultural Sciences and Veterinary Medicine Cluj-Napoca 400372 Romania; 28 Section for Parasitology and Aquatic Pathobiology, Department of Veterinary and Animal Sciences, University of Copenhagen DK-1870 Frederiksberg C Denmark; 29 Department of Zoology and Fisheries, Faculty of Agrobiology, Food and Natural Resources, Czech University of Life Sciences Prague Kamycka 129 165 00 Prague Suchdol Czech Republic; 30 Institute of Parasitology of the Slovak Academy of Sciences Kosice 040 01 Slovakia; 31 Institute for Parasitology and Tropical Veterinary Medicine, Freie Universität Berlin Robert-von-Ostertag-Str. 7–13 14163 Berlin Germany

**Keywords:** Anthelmintic resistance, Ruminants, Europe, Gastrointestinal nematodes, Liver fluke, Prevalence, Maps, Database

## Abstract

Helminth infections are ubiquitous in grazing ruminant production systems, and are responsible for significant costs and production losses. Anthelmintic Resistance (AR) in parasites is now widespread throughout Europe, although there are still gaps in our knowledge in some regions and countries. AR is a major threat to the sustainability of modern ruminant livestock production, resulting in reduced productivity, compromised animal health and welfare, and increased greenhouse gas emissions through increased parasitism and farm inputs. A better understanding of the extent of AR in Europe is needed to develop and advocate more sustainable parasite control approaches. A database of European published and unpublished AR research on gastrointestinal nematodes (GIN) and liver fluke (*Fasciola hepatica*) was collated by members of the European COST Action “COMBAR” (Combatting Anthelmintic Resistance in Ruminants), and combined with data from a previous systematic review of AR in GIN. A total of 197 publications on AR in GIN were available for analysis, representing 535 studies in 22 countries and spanning the period 1980–2020. Reports of AR were present throughout the European continent and some reports indicated high within-country prevalence. Heuristic sample size-weighted estimates of European AR prevalence over the whole study period, stratified by anthelmintic class, varied between 0 and 48%. Estimated regional (country) prevalence was highly heterogeneous, ranging between 0% and 100% depending on livestock sector and anthelmintic class, and generally increased with increasing research effort in a country. In the few countries with adequate longitudinal data, there was a tendency towards increasing AR over time for all anthelmintic classes in GIN: aggregated results in sheep and goats since 2010 reveal an average prevalence of resistance to benzimidazoles (BZ) of 86%, macrocyclic lactones except moxidectin (ML) 52%, levamisole (LEV) 48%, and moxidectin (MOX) 21%. All major GIN genera survived treatment in various studies. In cattle, prevalence of AR varied between anthelmintic classes from 0–100% (BZ and ML), 0–17% (LEV) and 0–73% (MOX), and both *Cooperia* and *Ostertagia* survived treatment. Suspected AR in *F. hepatica* was reported in 21 studies spanning 6 countries. For GIN and particularly *F. hepatica*, there was a bias towards preferential sampling of individual farms with suspected AR, and research effort was biased towards Western Europe and particularly the United Kingdom. Ongoing capture of future results in the live database, efforts to avoid bias in farm recruitment, more accurate tests for AR, and stronger appreciation of the importance of AR among the agricultural industry and policy makers, will support more sophisticated analyses of factors contributing to AR and effective strategies to slow its spread.

## Abstract (fr)

Les helminthes sont omniprésents dans les systèmes de production de ruminants au pâturage et sont responsables de coûts et de pertes de production importants. La résistance aux anthelminthiques (RA) des parasites est maintenant répandue dans toute l’Europe, bien qu’il existe encore des lacunes dans nos connaissances dans certaines régions et certains pays. La RA est une menace majeure pour la pérennité de la production moderne de ruminants, en diminuant la productivité, en compromettant la santé et le bien-être des animaux, et en augmentant les émissions de gaz à effet de serre au travers d’une augmentation du parasitisme et des intrants agricoles. Une meilleure compréhension de l’étendue de la RA en Europe est nécessaire pour développer et préconiser des approches de lutte antiparasitaire plus durables. Une base de données intégrant des informations publiées et non publiées en Europe concernant la RA des nématodes gastro-intestinaux (NGI) et de la douve du foie (*Fasciola hepatica*) a été compilée par les membres de l’action européenne COST « COMBAR » (« Combattre la résistance aux anthelminthiques chez les ruminants ») et combinée avec les données d’une précédente étude systématique concernant la RA des NGI. Au total, 197 publications sur la RA des NGI étaient disponibles pour analyse, représentant 535 études dans 22 pays et couvrant la période 1980–2020. Des signalements de RA étaient présents sur tout le continent européen et certains rapports indiquaient une forte prévalence nationale. Les estimations heuristiques pondérées par la taille de l’échantillon de la prévalence de la RA en Europe sur toute la période d’étude, stratifiées par classe d’anthelminthiques, variaient de 0 à 48 %. La prévalence régionale (nationale) estimée était très hétérogène, variant entre 0 % et 100 % selon le secteur de l’élevage et la classe d’anthelminthique, et augmentait généralement avec les efforts de recherche dans le pays. Dans les quelques pays disposant de données longitudinales adéquates, il y avait une tendance à l’augmentation de la RA au fil du temps pour toutes les classes d’anthelminthiques des NGI : les résultats agrégés chez les ovins et caprins depuis 2010 révèlent une prévalence moyenne de résistance aux benzimidazoles (BZ) de 86 %, aux lactones macrocycliques sauf moxidectine (ML) de 52 %, au lévamisole (LEV) de 48 % et à la moxidectine (MOX) de 21 %. Tous les genres principaux de NGI ont survécu au traitement dans diverses études. Chez les bovins, la prévalence de la RA variait selon les classes d’anthelminthiques de 0 à 100 % (BZ et ML), 0 à 17 % (LEV) et 0 à 73 % (MOX), et *Cooperia* et *Ostertagia* ont survécu aux traitements. Une RA suspectée chez *F. hepatica* a été signalée dans 21 études portant sur 6 pays. Pour les NGI, et encore plus pour *F. hepatica*, il y avait un biais d’échantillonnage en faveur des exploitations individuelles suspectées de RA, et l’effort de recherche était biaisé vers l’Europe occidentale et en particulier le Royaume-Uni. La capture continue des résultats futurs dans la base de données, en direct, les efforts pour éviter les biais dans le recrutement des exploitations, des tests plus précis pour la RA et une meilleure appréciation de l’importance de la RA parmi l’industrie agricole et les décideurs politiques, soutiendront des analyses plus sophistiquées des facteurs contribuant à la RA, et des stratégies efficaces pour ralentir sa propagation.

## Introduction

Livestock production is estimated to contribute on average 40% of agricultural production value worldwide (~60% in high income countries, ~30% in low- and middle-income countries) [45]. The infection of ruminant livestock with helminth parasites, such as gastrointestinal nematodes (GIN, primarily trichostrongylids) and liver fluke (primarily *Fasciola hepatica*) threatens the profitability and sustainability of livestock production. Helminths affect growth [35], productivity [9] and reproductive success [46], and threaten the ability of livestock farmers to maintain high health and welfare standards. Helminth infections are predicted to cost the European ruminant livestock industry €1.8bn annually [9]. Furthermore, helminth infections may increase greenhouse gas (GHG) emissions associated with ruminant livestock production; in Scotland, liver fluke infection is predicted to increase emissions from milk production by ~10% [1] and GIN infection is predicted to increase GHG emissions intensity in sheep systems by up to 30% [16, 23, 29, 47]. Therefore, effective and sustainable helminth control is essential in order to maintain high levels of productivity and welfare, and limit the contribution of the livestock sector to agricultural GHG emissions.

Ruminant helminth control is heavily reliant on the use of anthelmintic products such as benzimidazoles (BZ; e.g. albendazole, triclabendazole), levamisoles (LEV) and macrocyclic lactones (ML; e.g. ivermectin, eprinomectin, moxidectin) to maintain infections below levels that can cause clinical and sub-clinical disease. However, Anthelmintic Resistance (AR) in GIN has been reported worldwide in multiple nematode species, against most anthelmintic classes and sometimes simultaneously multiple different classes [27, 42, 49] and there is increasing evidence that resistance in liver fluke (*F. hepatica*) may also be widespread (e.g. [3, 18, 25]). The annual cost of AR in Europe has been estimated at €38 million and this cost is predicted to grow because of the increasing spread of resistant populations as well as occurrence of helminths resistant to multiple anthelmintic classes [9].

Newer, more sustainable, approaches to helminth control, which reduce the frequency of anthelmintic treatments (therefore, reducing selection pressure for resistance) while maintaining high productivity levels are available. These approaches include Targeted Selective Treatment (TST), based on individual animal health or production parameters [8, 34], or Targeted Treatment (TT), based on regular seasonal factors and/or diagnostic measures to inform treatment decisions at group or flock level [8]. Regional industry bodies invest significant resources into advocating for these sustainable approaches (e.g. in the UK, Sustainable Control of Parasites in Sheep (https://www.scops.org.uk) and Control of Worms Sustainably (https://www.cattleparasites.org.uk), yet farmers continue to implement AR-selective practices, such as moving sheep to low challenge or “clean” pastures immediately after treatment and treating ewes and lambs more frequently than necessary [24]. Confirmation of AR and a perceived risk of AR were identified as having a significant positive effect on the uptake of sustainable parasite control practices by Scottish sheep farmers [24]. Similar attitudes were found in the Belgian cattle industry, where a positive attitude towards anthelmintics was a barrier to the uptake of sustainable control practices [51]. This suggests that increasing farmer awareness of the risk associated with AR may promote the uptake of sustainable control practices.

A systematic review of reports of AR in European ruminant livestock confirmed that AR is spatially widespread, but also highlighted limitations in the available data, due to sample selection bias, a lack of georeferenced data to allow robust spatial analyses, and a number of countries for which no data existed [42]. In 2017, the EU COST Action COMBAR (Combatting Anthelmintic Resistance in Ruminants; https://www.combar-ca.eu) established a consortium of researchers from 26 countries across Europe. The consortium aims to advance research on the prevention of AR in helminth parasites of ruminants and disseminate current knowledge among all relevant stakeholders through improving diagnosis, understanding the socio-economic aspects of infection and decision making, and developing innovative, sustainable control methods. As part of this, and to encourage the uptake of COMBAR outputs and recommendations for sustainable control of helminth parasites, an up-to-date synthesis of current AR research is needed.

In this study, we draw on the collective expertise and data held by the COMBAR consortium to address the limitations of AR research identified previously, by collating published and unpublished data on AR in Europe that can be used to support advocacy for sustainable control programmes and to target future research efforts where they are needed most.

## Methods

### Systematic review protocol

This review was performed and is reported according to the Preferred Reporting Items for Systematic Reviews and Meta-Analyses (PRISMA) [36]: (1) preparation of a database search to detect potentially related articles, (2) assessment of the relevancy of papers, (3) evaluation of quality, and (4) data extraction.

#### Data collection and processing

A standardised spreadsheet model was populated with data from the previous systematic review [42]. Members of the COMBAR consortium were then invited to populate the spreadsheet with published and unpublished data for completed research for their respective country and surrounding countries, if no representative was present in the consortium (e.g. Finland). The database consequently included reports from 1980–2020. In adherence to the PRISMA guidelines, contributing authors were asked to search electronic databases (e.g. PubMed, ISI Web of Science) and unpublished studies with no language or date restrictions. Data requested included the host species, region, number of farms tested, anthelmintic drug class and active compound tested, whether AR was detected (1) or not (0), the proportion of farms tested where AR was detected, reference, DOI (if applicable) and publication type (unpublished, peer-reviewed paper, Master or PhD thesis, report, or conference abstract/proceeding). Each publication was screened for eligibility (i.e. a study reporting the presence or absence of AR in ruminant livestock helminths on the European continent), duplicates eliminated, and the eligible studies fully reviewed. The data were then edited for consistency and checked against the relevant publication (if applicable) by the lead author, before being checked a second time by participating consortium members. The consortium member responsible for the final check is recorded in the database.

Each publication or unpublished dataset, hereafter referred to as “publication”, was given a unique identifying number. Within each publication, multiple anthelmintic classes and livestock classes tested were entered as separate entries, hereafter referred to as “studies”. Therefore, one “publication” may contain several “studies”. When a publication presented separate data for different regions within the same country, these data were split into separate rows (studies) in the database (e.g. [20]). When a publication presented data together for different regions within the same country, the data were entered as a single row (study) in the database (e.g. [17]).

For GIN, data were stratified primarily by host species and anthelmintic class, with the exception of the ML, which were divided into avermectins (e.g. ivermectin) and milbemycins (i.e. moxidectin, MOX). Hereafter, MOX is analysed separately to the other ML. Other classes were benzimidazoles (BZ), imidazothiazoles, i.e. levamisole (LEV), amino-acetonitrile derivatives, i.e. monepantel (MPTL), and salicylanilides, i.e. closantel (CLOS). Studies testing more than one anthelmintic simultaneously in the same animals were entered as combination treatments (COMB), except where the combination involved MOX and ivermectin; in this case the anthelmintic class was entered as MOX due to its relatively high efficacy against some ivermectin-resistant populations of GIN, and observations that the extent of cross-resistance is greater in ivermectin than MOX-resistant populations (reviewed by [39]).

For *F. hepatica*, data were stratified by anthelmintic compound, rather than class, in order to differentiate between the activity of triclabendazole (TBZ) and albendazole (ALB), which have different efficacy profiles depending on the age of the fluke [15]. Data for cattle and sheep were combined for analyses as *F. hepatica* are genetically similar between host species [4].

#### Inclusion criteria

Due to significant upcoming changes in protocols for detecting AR [26], inclusion criteria were pragmatic and less strict than in [42], and all publications investigating AR in livestock in Europe were eligible for inclusion. The authors’ classification of resistance was accepted in each case, regardless of method used and level of detail provided. Although this may result in the inclusion of publications that do not conform to the previous guidelines on detecting AR in ruminants, it allows the inclusion of unpublished reports (e.g. conference proceedings and oral presentations), where detailed information on the methodology (*in vivo*; Faecal Egg Count Reduction Test (FECRT) and Controlled Efficacy Test (CET), *in vitro,* and molecular methods) is unavailable, therefore increasing the timeliness of the analysis and potentially reducing limitations such as regional publication bias. Some misclassification of resistance is consequently expected in both directions, but the increased number of studies adds power to analysis of trends.

### Data analysis

#### Prevalence, uncertainty and heterogeneity

Data analysis was completed in R v3.6.1 [40]. Figures were prepared using the base R, “ggplot2” [54] and “ggpubr” [28] packages. Tables were prepared using the sjPlot R package [32]. Maps were prepared using the “rnaturalearth” [48], “rgeos” [5] and “ggspatial” [13] packages.

GIN AR prevalence for each anthelmintic class and ruminant species were estimated from the reported prevalences and were weighted by sample size (number of farms in each study) to give a weighted mean (${\bar{x}}_{w}$), using the “weighted.mean” function in R. Analyses were performed within each country with data, and overall across Europe. Corresponding sample size weighted standard deviations were estimated using Equation (1), where *x* = the proportion of farms with AR, *w* = weights (sample size divided by the sum of all samples):

$${\;\sigma }_{w}=\;\sum (w\;\times (x-{\bar{x}}_{w}{)}^{2}).$$

Heterogeneity in AR prevalence estimates between studies (aggregated by anthelmintic class and host species) was estimated using the *I*^2^ statistic [21], calculated as described by Neyeloff et al. [38], with the exception that a small value was added to the prevalence estimates (1 × 10^−7^) to allow variance to be calculated as input for Cochran’s Q statistic when estimated prevalence was zero. Negative *I*^2^ values were set to zero [21], as well as any instances where all studies found zero prevalence. *I*^2^ values were not estimated where only one study existed. Higher *I*^2^ values indicate higher levels of heterogeneity.

To evaluate the impact of sample selection bias in studies with small sample sizes (assuming that those with very small sample sizes preferentially selected farms with suspected AR), sample size-weighted mean prevalences, standard deviations and *I*^2^ were also estimated using only studies investigating 10 or more farms.

Similar analyses were not completed for *F. hepatica* due to insufficient studies.

#### Spatial bias in research effort

Qualitative assessment of spatial bias in AR research was made by mapping the number of publications per ruminant species per country. Quantitative assessment of spatial bias in AR research (GIN only) was made by relating the number of studies per country and the research budget or cost of helminth infection per country using Spearman rank correlation (“cor.test*”* function in R) and estimates for the cost of helminth infections and research budget for projects starting after 01/01/2008 and ending before 31/12/2017 [9].

To compare the estimated level of AR between countries alongside the relative confidence that can be placed in that estimate, an index was compiled for each, as follows:

$$\mathrm{Level}\;\mathrm{of}\;\mathrm{AR}=\;\sum_{1}^{n}p+\;n_{a},$$

where *p* is the sample-size weighted prevalence of AR (i.e. the proportion of farms testing positive) for each active compound, and *n*_*a*_ is the number of actives to which resistance was documented to date. For GIN, prevalence for up to four actives was included: BZ, LEV, ML, and MOX separately from other MLs as a special case (as detailed above). MPTL was not included in the prevalence part of the equation as it has been tested only rarely to date, and the same applies for the narrow-spectrum active sulphonamide, CLOS. Both, however, were added to *n*_*a*_ in countries in which cases of resistance had been confirmed. Thus, for instance, a country with reports of AR involving BZ and ML with sample-size weighted prevalence of 0.5 and 0.1, would score (0.5 + 0.1 + 2 = 2.6), while another reporting resistance to BZ, LEV, ML, MOX and MPTL with prevalence, respectively of 1, 0.7, 0.8, 0.4 and 0.1, would score (1 + 0.7 + 0.8 + 0.4 + 5 = 7.9). For liver fluke, seven actives were included in both *p* and *n*_*a*_ (see Results).

Confidence was not estimated from prevalence; rather, it is an index of research effort, taking into account the depth and breadth of investigation into the AR situation nationally:

$$\mathrm{Confidence}\;\left(\mathrm{research}\;\mathrm{effort}\right)=\;\frac{\log_{10}\left(S\right)}{\max\left(\log_{10}\left(S\right)\right)}+\frac{\log_{10}\left(F\right)}{\max\left(\log_{10}\left(F\right)\right)}+A,$$

where, for each country, *S* is the number of separate publications (or unpublished reports) in the database, *F* the total number of farms included, and *A* the total number of anthelmintic classes investigated. *F* is the sum of the total number of farms used per study, regardless of how many anthelmintics were assessed on each, to prevent double counting. Both *S* and *F* were log_10_-transformed to stabilise variance, and scaled to the maximum value across countries (i.e., divided by the highest national value), to provide a measure of the relative research effort among countries in the database at the time of the analysis. One publication from Ireland included data from 4211 farms but used pooled faecal samples collected by farmers: for this case, the number of farms was divided by an arbitrary value of 10 before entry into the equation, to avoid unfair comparison with studies using full FECRT. The confidence index itself is arbitrary, but standardised across countries, and aims to assess the relative extent to which AR has been thoroughly investigated, to aid interpretation and prioritisation of future research.

Results of *Level* versus *Confidence* (*= Research effort*) were plotted for each country: separately for GIN in either sheep, goats or cattle, and for fluke for all host species combined. Data manipulation and analysis were completed using Excel (Microsoft, USA).

#### Change in anthelmintic resistance over time

Anthelmintic Resistance is a dynamic process, evolving as helminth populations experience repeated treatment, and as farmers change to more effective anthelmintics when treatment failure is recognised. Research on AR is also dynamic, often responding initially to reports of treatment failure on particular farms, before expanding to prospective surveys, with highly variable selection criteria, and assessing additional anthelmintic groups as they become available or fall under suspicion of failure. Because of this, prevalence data averaged over a long period are unlikely to accurately capture the current situation in any given country, or trends in AR over time.

To address these confounders, change in prevalence was evaluated over time. Reports of AR on single farms were excluded, since these are often conducted in response to suspected treatment failure. Prevalence was transformed to arcsine of the square root of prevalence in order to stabilise the variance around values close to 0 or 1. The relationship between year and prevalence was then tested using Pearson and Spearman rank correlation.

In a second stage analysis, the data set was reduced to countries that reported at least three studies in different years, to further decrease the influence of responsive investigations and to capture change over time within countries. Sample-size adjusted prevalence, while arguably giving the fairest indication of how common AR is among farms in a given sample set, can result in bias: for example, if larger surveys are conducted as concern over resistance rises over time, more recent data will dominate overall prevalence estimates. For this reason, prevalence from individual studies was averaged over each decade without adjusting for sample size. Studies in sheep and goats were combined to increase power, given that helminth species are largely shared between these hosts. Italy was excluded because its combination of low AR prevalence and high level of reporting in recent years (19/20 reports in sheep and goats coming after 2007) strongly affected the overall trajectory across countries, and a single large study in the UK focusing only on *Nematodirus battus* was also excluded. Initially, average prevalence was plotted for each anthelmintic group for each decade, to visualise trends in AR over time. The database was then searched separately for each anthelmintic group in GIN in sheep, goats and cattle, and fluke in all hosts. For each country meeting these criteria (including Italy), correlation between prevalence and year was assessed as above. Where a significant correlation was confirmed, linear regression was conducted to evaluate the rate of change in AR prevalence over time.

#### Qualitative analysis of factors influencing research on anthelmintic resistance

The main relevant influences that might affect research on AR were identified by SWOT (Strengths, Weaknesses, Opportunities, and Threats) analysis, to inform further AR research. The SWOT template was drafted by the lead authors and circulated for input by all co-authors.

## Results

The database used in these analyses is provided as Supplementary Material. A live version of the database is also freely available on the Open Science Framework [43] and submissions to update this website are invited via the COMBAR website [11]. This version will be updated periodically with additional data submitted by contributors and is made available for reuse on a General Public license (https://www.gnu.org/licenses/gpl-3.0.html).

### Gastrointestinal nematodes

**Figure 1:**
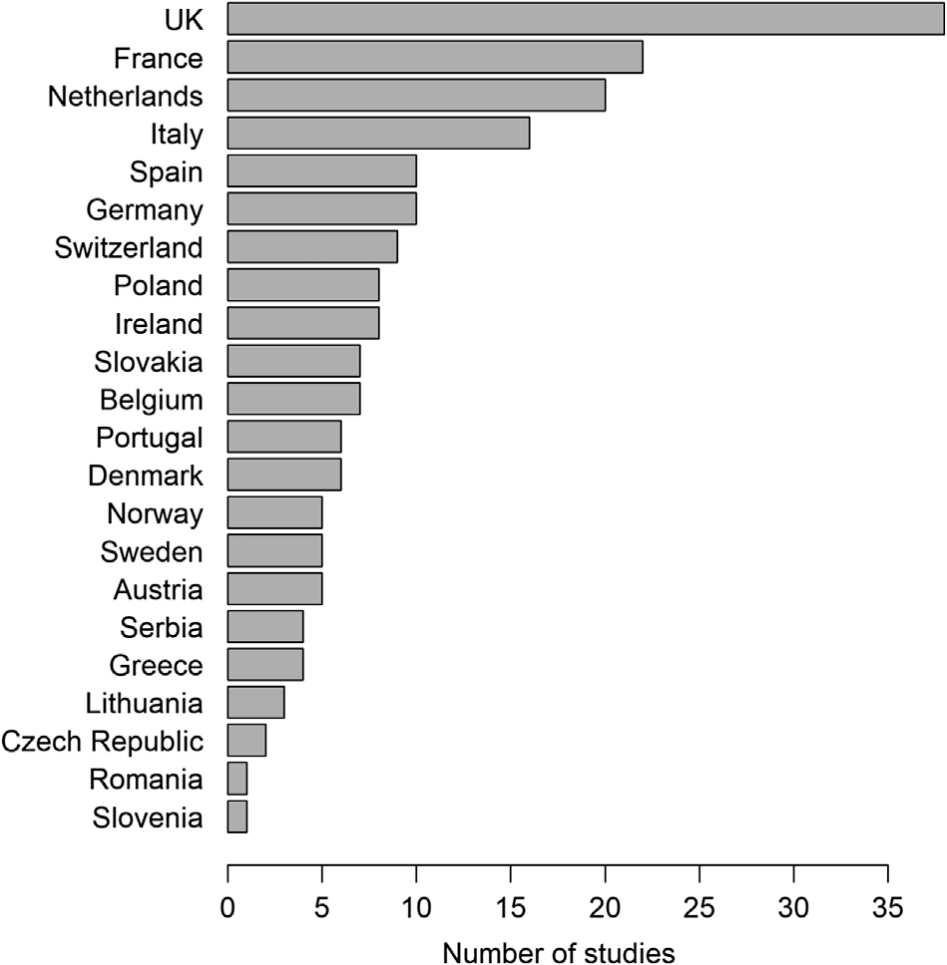
Number of publications investigating anthelmintic resistance in gastro-intestinal nematodes reported from each country and included in the database.

In all, 197 publications were available for analysis, representing 535 studies, 22 countries and spanning the period 1980–2020 (Fig. 1 and https://www.parasite-journal.org/10.1051/parasite/2020062">Fig. S1). The majority of studies investigated AR in GIN on single farms (137/535 studies; median number of farms = 5, range = 1–550). 23.4% (*n* = 47) of the “publications” were unpublished.

**Figure 2:**
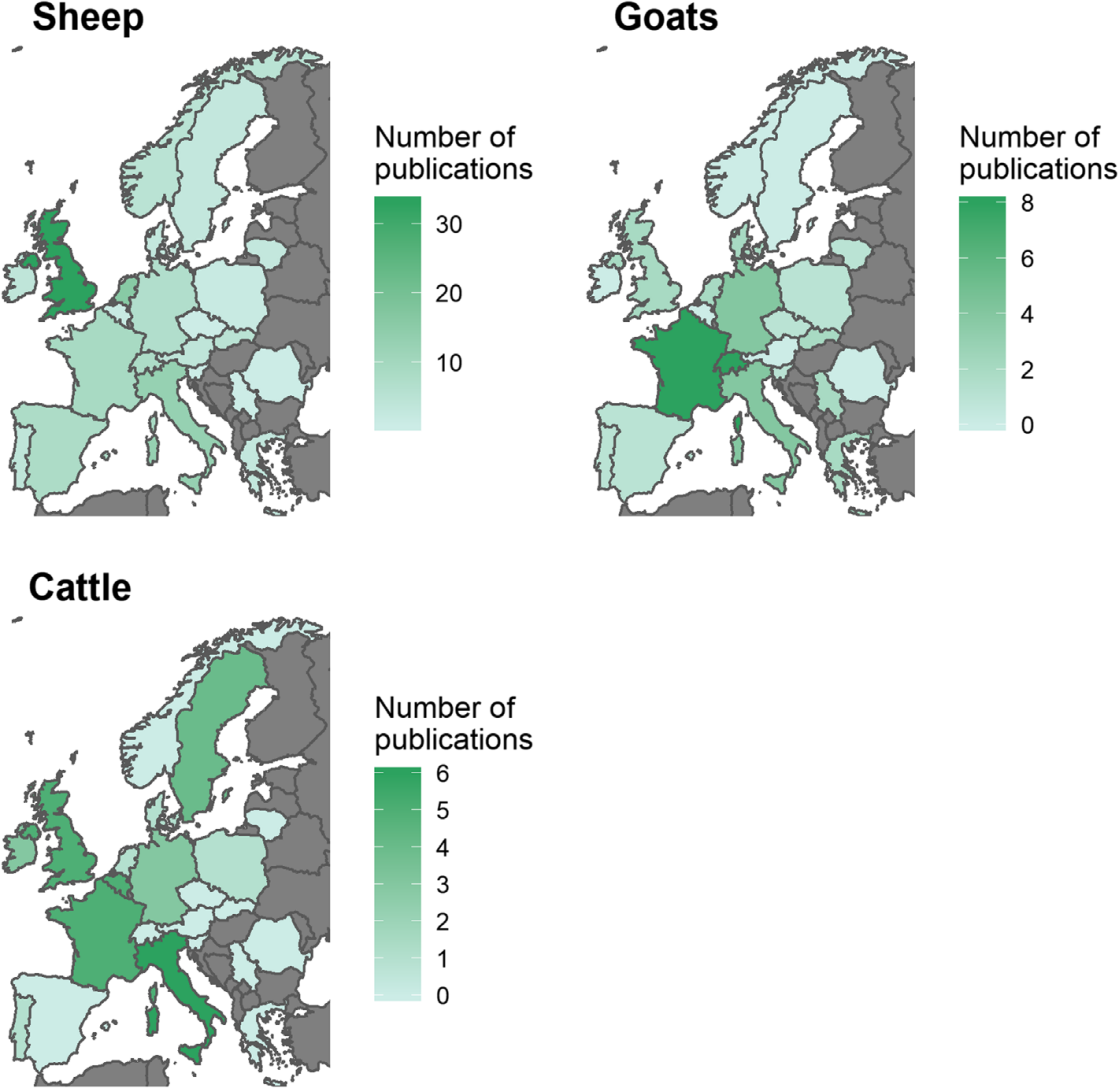
Number of publications investigating anthelmintic resistance in gastrointestinal nematodes for each ruminant host species. Green colours represent the number of publications. Grey indicates no data. Note the different symbology ranges for each ruminant species. Variation within countries is not shown.

There was a regional research bias towards Western Europe and especially the UK, while Eastern European countries were relatively underrepresented in the body of AR research (Figs. 1 and 2; the four countries with the fewest publications were located in Eastern Europe). The number of studies per country was not correlated with the research budget reported by Charlier et al. [9] (*p* = 0.49) but was positively correlated with the economic cost of helminth infections estimated in the same study (*S* = 177.95, *ρ* = 0.68, *p* = 0.005).

**Figure 3:**
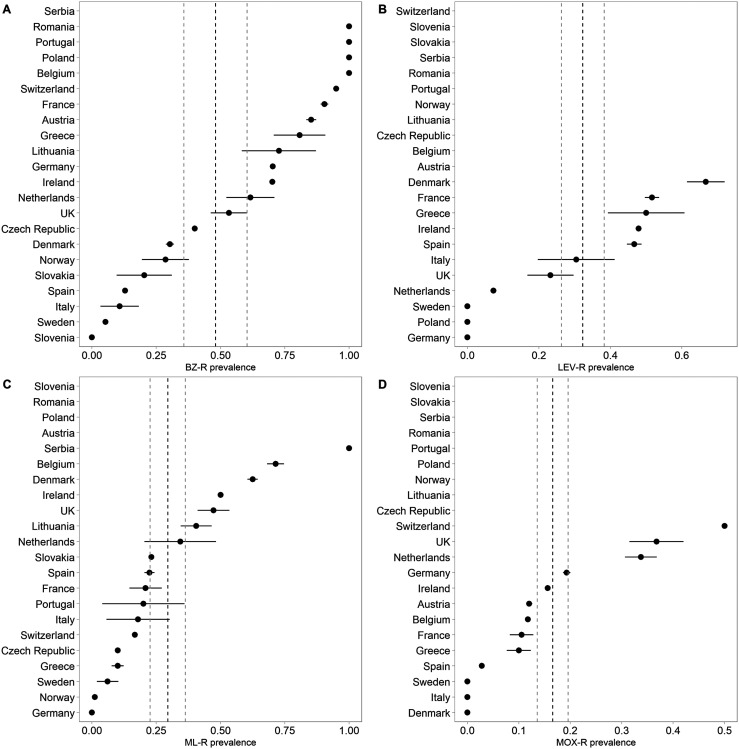
Estimated prevalence of resistance against the benzimidazoles (BZ), levamisole (LEV), avermectins (ML; macrocyclic lactones), and moxidectin (MOX) in gastrointestinal nematodes in sheep. Points and whiskers represent the weighted prevalence estimate and standard deviation, respectively. The weighted prevalence and standard deviation across all studies are represented by the dashed black and grey vertical lines, respectively. Note that points without whiskers represent single studies, for which standard deviations could not be estimated (i.e. they do not represent points which the prevalence is known with a high level of confidence). Corresponding figures for goats and cattle, and for sheep, goats and cattle using only studies with *n* > 9 can be found in the Supplementary Material.

Reports of AR were widespread throughout Europe, particularly to BZ (Figs. 3 and https://www.parasite-journal.org/10.1051/parasite/2020062">Figs. S2–https://www.parasite-journal.org/10.1051/parasite/2020062">S4). AR was most widely reported in sheep, whereas AR in cattle and goats was understudied in many regions, indicated by the number of countries with no data available (https://www.parasite-journal.org/10.1051/parasite/2020062">Fig. S4).

Sample-size weighted prevalence estimates ranged between 0% and 100%, depending on the country and anthelmintic class (Table 1; Fig. 3 and https://www.parasite-journal.org/10.1051/parasite/2020062">Figs. S2–https://www.parasite-journal.org/10.1051/parasite/2020062">S3). However, there was considerable heterogeneity between studies (Table 1). Overall, in sheep and goats, AR prevalence was highest against BZ, whereas in cattle AR prevalence was highest against ML, including MOX. Resistance was reported in sheep against all classes of anthelmintic except derquantel-abamectin in combination, which was not tested. No resistance was reported against MOX or MPTL in goats (only one and two studies, respectively, tested these anthelmintics in goats). Only one study tested MPTL in cattle, and resistance was not detected. CLOS was not tested in goats nor cattle (Table 1). The difference between prevalence estimates using all studies and studies with ten or more farms was negligible (Table 1), and *I*^2^ was similar (Table 1). Therefore, unless otherwise stated, all prevalence estimates shown used all studies.

**Table 1:** Mean (SD in parenthesis) sample size-weighted prevalence of anthelmintic resistance stratified by anthelmintic class and host livestock sector. BZ = benzimidazole; LEV = levamisole; ML = avermectins; MOX = moxidectin; MPTL = monepantel; CLOS = closantel; – = no data available. *I*^2^ is provided as a measure of heterogeneity (high *I*^2^ = high heterogeneity between studies). Number of studies (number of publications in parenthesis) indicates the number of groups tested, which may be >1 per publication. For example, some publications provide results for multiple regions. Columns labelled “*n* > 9” used only studies with 10 or more farms, while columns labelled “all” used all data.

Anthelmintic	Host	Prevalence (all)	Studies (all)	*I*^2^ (all)	Prevalence (*n* > 9)	Studies (*n* > 9)	*I*^2^ (*n* > 9)
BZ	Sheep	0.48 (0.12)	123 (94)	0.93	0.47 (0.12)	64 (52)	0.96
	Cattle	0.08 (0.04)	15 (12)	0	0.08 (0.04)	7 (6)	0.33
	Goats	0.51 (0.17)	31 (29)	0.86	0.52 (0.16)	12 (11)	0.94
LEV	Sheep	0.32 (0.06)	43 (40)	0.88	0.31 (0.05)	19 (18)	0.94
	Cattle	0.12 (0.01)	4 (4)	0	0.18 (0)	1 (1)	–
	Goats	0.2 (0.04)	11 (11)	0.68	0.21 (0.03)	4 (4)	0.54
ML	Sheep	0.29 (0.07)	83 (61)	0.79	0.29 (0.06)	30 (28)	0.91
	Cattle	0.32 (0.1)	31 (24)	0.78	0.25 (0.08)	10 (9)	0.89
	Goats	0.44 (0.18)	27 (24)	0.79	0.43 (0.18)	7 (7)	0.94
MOX	Sheep	0.17 (0.03)	36 (35)	0.68	0.17 (0.03)	13 (13)	0.87
	Cattle	0.27 (0.1)	9 (6)	0.50	0.23 (0.09)	4 (3)	0.70
	Goats	0.01 (0.01)	7 (7)	0	0 (0)	1 (1)	–
MPTL	Sheep	0.05 (0.03)	10 (10)	0	0.02 (0)	3 (3)	0
	Cattle	0 (0)	1 (1)	–	–	0 (0)	–
	Goats	0 (0)	2 (2)	0	0 (0)	1 (1)	–
CLOS	Sheep	0.25 (0)	2 (2)	0.75	0.27 (0)	1 (1)	–
	Cattle	–	0 (0)	–	–	0 (0)	–
	Goats	–	0 (0)	–	–	0 (0)	–

Information on AR in different species of GIN was limited by variation in the extent to which species surviving treatment were characterised, and the methods used to assess and report this. Genera identified in post-treatment samples included *Teladorsagia, Haemonchus, Trichostrongylus*, *Cooperia* and *Nematodirus* in sheep and/or goats, and *Ostertagia* and *Cooperia* in cattle.

### Fasciola hepatica

Anthelmintic Resistance in *F. hepatica* was reported in 21 publications, spanning six countries (Ireland, Italy, Netherlands, Spain, Sweden and UK). TBZ resistance was reported in the UK, Ireland, Spain and the Netherlands. ALB resistance was reported in Sweden and Spain. CLOS resistance was reported in Sweden, and clorsulon resistance was reported in Spain. No resistance was observed in Italy. The majority of publications investigated AR on individual farms, with the exception of two larger studies. Kamaludeen et al. [25] identified TBZ resistance in 80% of 26 farms in the UK. Holzhauer et al. [22] identified TBZ resistance in 12% of 26 farms in the Netherlands selected due to their high risk of AR. Most individual case reports of TBZ resistance in *F. hepatica* originated from the Netherlands and were reported on 94 farms by the Royal Animal Health Service between 1998 and 2003 (https://www.gddiergezondheid.nl).

### Level of AR in relation to research effort

**Figure 4:**
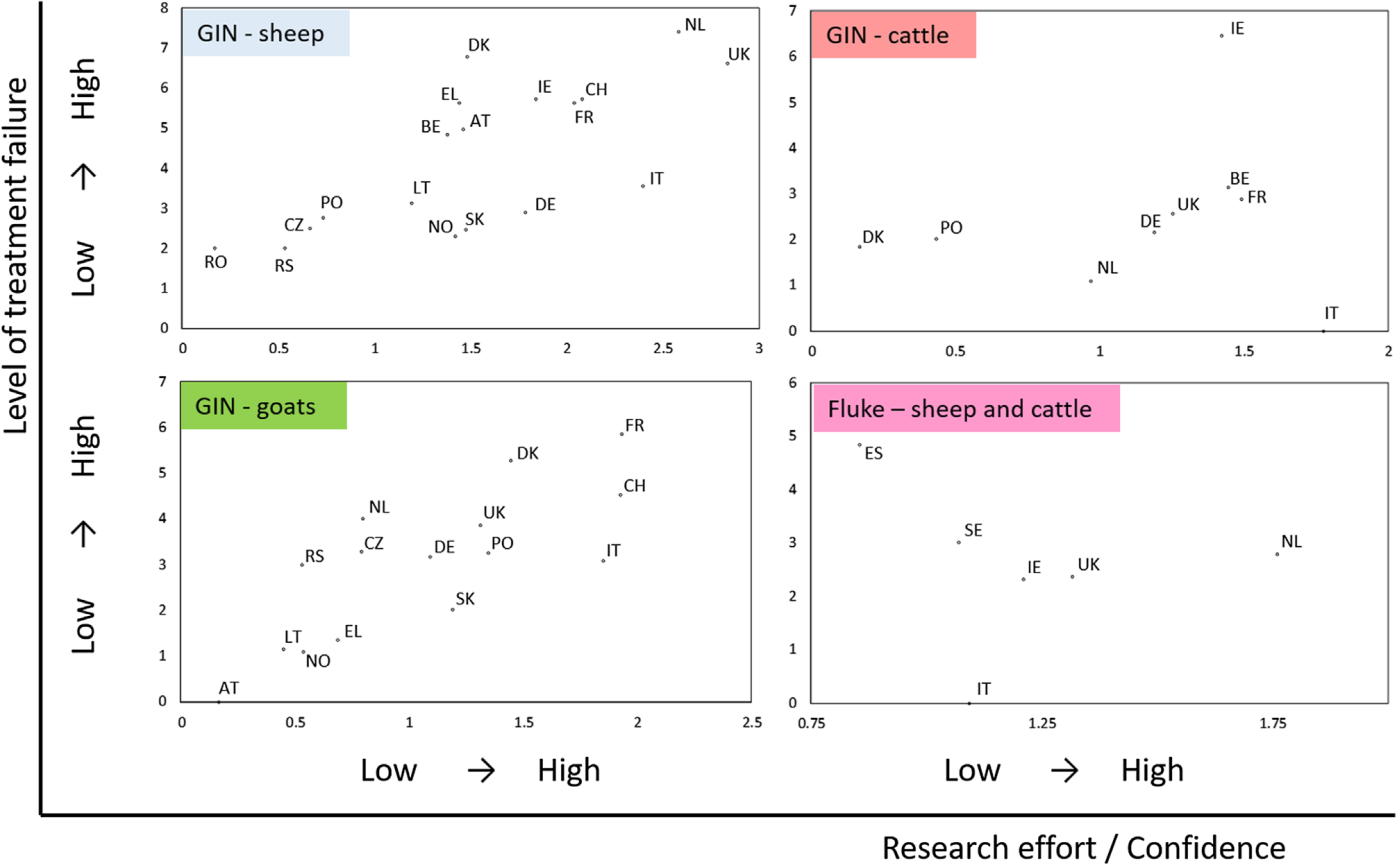
Level of anthelmintic resistance in each country, using a composite index across all drugs tested, in relation to research effort (= confidence) as a function of number of studies, farms and anthelmintics investigated. See methods for details of indices. Note that horizontal scales are different for each panel. For gastrointestinal nematodes in sheep in Ireland, the confidence score was modified to take account of a single large study that assessed treatment efficacy through pooled faecal sampling by farmers at and after treatment [54]. GIN = Gastrointestinal nematodes. Country abbreviations: AT = Austria, BE = Belgium, CH = Switzerland, CZ = Czechia, DE = Germany, DK = Denmark, EL = Greece, ES = Spain, FR = France, IE = Ireland, IT = Italy, LT = Lithuania, NL = Netherlands, NO = Norway, PL = Poland, RO = Romania, RS = Serbia, SE = Sweden, SK = Slovakia, UK = United Kingdom.

For GIN in sheep and goats, the observed level of treatment failure correlated with research effort (*ρ*_18_ = 0.75, *p* = 0.001; and *ρ*_15_ = 0.79, *p* < 0.001, respectively), but this relationship was not apparent for studies of GIN in cattle (*p* = 0.46), or for fluke across host species (*p* = 0.40; Fig. 4). Across all host-parasite combinations, countries with low levels of observed AR also had low levels of confidence (research effort) attached to those estimates, as a result of limited number, size and/or breadth of studies. Only in a few countries (Germany and Italy for GIN in sheep, and Italy for GIN in goats and cattle) was a relatively low overall level of AR associated with relatively robust assessment. Even in these countries, higher prevalence of AR for some active compounds should be noted (Fig. 3 and https://www.parasite-journal.org/10.1051/parasite/2020062">Figs. S2–https://www.parasite-journal.org/10.1051/parasite/2020062">S3).

### Change in reported anthelmintic resistance over time

**Figure 5:**
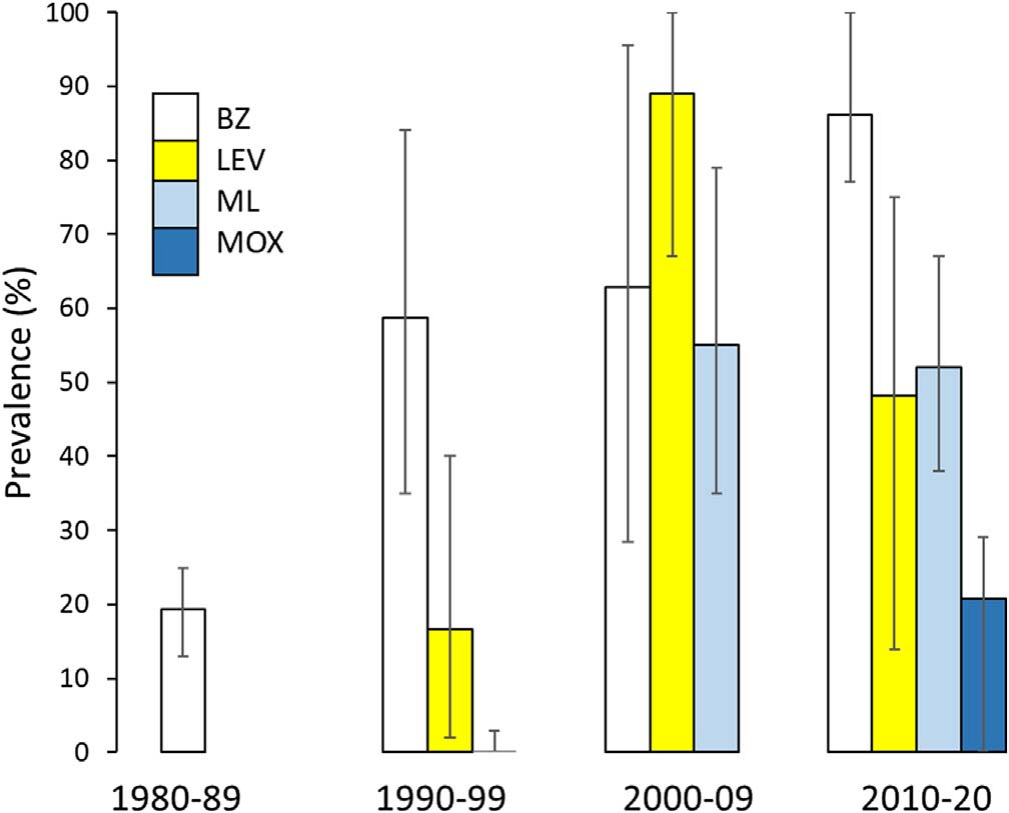
Average farm-level prevalence of anthelmintic resistance in gastrointestinal nematodes in sheep and goats, by decade. The arithmetic mean was taken of all reports including more than one farm, from countries with a minimum of three reports in different years, excluding Italy (see text). BZ = benzimidazoles, *n* = 64; LEV = levamisole, *n* = 14; ML = macrocyclic lactones excluding moxidectin, *n* = 23; MOX = moxidectin, *n* = 10. Error bars are standard deviations.

Prevalence of AR tended to increase over the 41 years included in the database (Fig. 5), gradually for BZ and more abruptly for ML (including MOX). Aggregated results in sheep and goats since 2010 returned average prevalence of resistance to benzimidazoles (BZ) of 86%, levamisole (LEV) 48%, macrocyclic lactones except moxidectin (ML) 52%, and moxidectin (MOX) 21%. Reports of farms with GIN resistant to LEV became more common between 1980 and 2009, but then decreased in 2010–2020. Taking reports from sheep, goats and cattle separately for each anthelmintic group, there was no significant correlation between farm-level prevalence of AR and year. In cattle, the prevalence of AR varied widely between anthelmintic classes from 0–100% (BZ and ML), 0–17% (LEV) and 0–73% (MOX). Only 6 of the 61 studies of cattle GIN in the database were conducted prior to 2009, however, and the median number of farms included was six, so the power to track change over time was very limited. Similarly, database entries for liver fluke comprised 34 studies from 18 sources, of which three were prior to 2009, and the median number of farms per study was one, precluding further exploration of temporal trends in AR.

**Figure 6:**
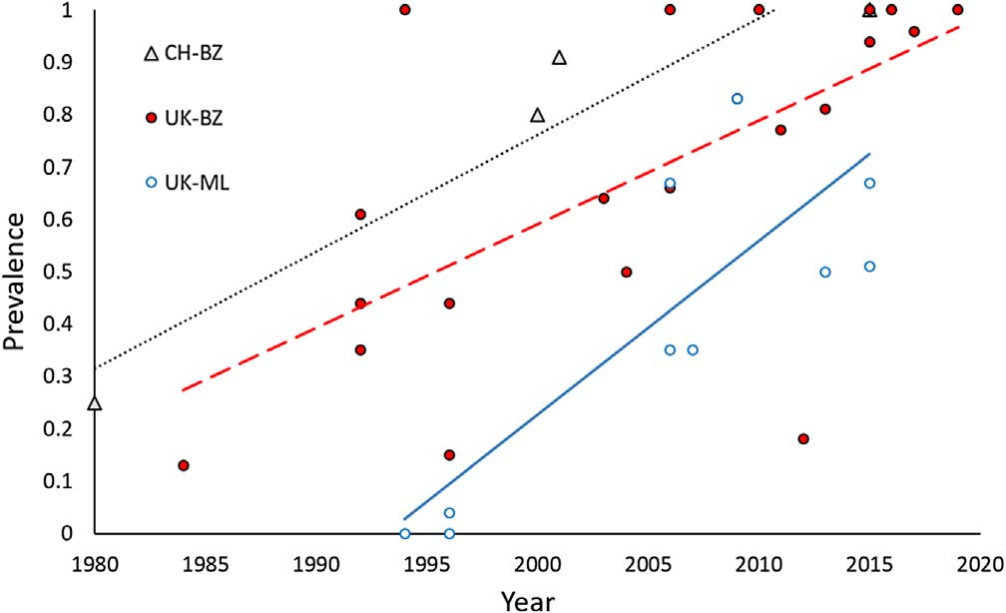
Change in the prevalence of anthelmintic resistance (= proportion of farms testing positive) for benzimidazoles (BZ) in Switzerland (CH; dotted black line) and the United Kingdom (UK; dashed red line), and macrocyclic lactones excluding moxidectin (MOX) in the United Kingdom (solid blue line). Only studies testing more than one farm were included. For regression equations, see https://www.parasite-journal.org/10.1051/parasite/2020062">Table S4(a–c). Data point for CH-BZ at (2016, 1) is partly concealed.

For GIN in sheep, six countries reported at least three studies in different years, excluding single-farm case reports: France (BZ), Italy (BZ, LEV, ML), the Netherlands (BZ, ML), Slovakia (BZ) Switzerland (BZ), and the United Kingdom (BZ, LEV, ML). No countries met this criterion for GIN in goats or cattle, or fluke. Further analysis of these 11 country-anthelmintic group combinations for GIN in sheep, on arcsine-transformed prevalence and with a Bonferroni-adjusted critical *p*-value of 0.0023, found significant correlations between year and farm-level prevalence of AR for BZ in Switzerland (*r*_p_ = 0.99, *p* < 0.001) and in the UK for BZ (*r*_p_ = 0.62, *p* = 0.002) and ML (*r*_p_ = 0.86, *p* = 0.001). Correlation in other countries was mostly positive but not significant. Linear regression of prevalence against year (Fig. 6; https://www.parasite-journal.org/10.1051/parasite/2020062">Table S4(a–c)) produced similar slopes for the rate of increase in BZ in Switzerland and the UK, equating to approximately 20% increase in prevalence per decade, and a faster rate of increase of 33% per decade for ML.

### SWOT analysis

The main relevant influences that might affect research on AR that were identified by SWOT analysis (Strengths, Weaknesses, Opportunities, and Threats; Table 2) included the strengths of existing consortia, lack of standardisation and continuity (in space and time) of data collection and surveillance, opportunities to share data and develop coordinated approaches in future, and potential public engagement, legislative and practical limitations.

**Table 2:** SWOT analysis for AR research in Europe. COMBAR = EU COST Action Combating Anthelmintic Resistance in Europe [37]; AR = Anthelmintic Resistance; STAR-IDAZ IRC = International Research Consortium on Animal Health.

Internal		External	
Strengths	Weaknesses	Opportunities	Threats
European initiative endorsed by all COMBAR members	Data on AR not collected on a constant basis (non-continuous and non-consistent data collection)	Real-time data sharing including a live database on AR in ruminant livestock	Insufficient public awareness of AR (compared to antimicrobial resistance)
Consistency and compliance with the priorities of the STAR-IDAZ IRC on animal health	Lack of harmonised tests for field and laboratory detection of AR to allow early reaction	Development of coordinated European surveillance system of AR.	The pattern in the AR maps could be misleading (black-box effect)
		Disseminate knowledge and promote the use of standardised methods for determining AR	
Live database compiled by regional experts with published and unpublished data	Fragmented surveillance systems and data biased towards Northern European countries and towards GIN	Striving to obtain precise location of AR cases	Data protection legislation may hamper the availability of georeferenced data to allow robust spatial analyses of AR
Operate in real time, providing maps, tables and reports	Spatial sampling bias can limit the analysis and interpretation of AR data	Rapid, responsive, efficient, and cost-effective planning of AR surveys	Confounding factors not related to AR may not be taken into consideration

## Discussion

In the present study, we reviewed the published and unpublished literature on AR in ruminant livestock in Europe from 1980 to 2020 to strengthen the current evidence base of this phenomenon at country and continental levels. A database of European AR research was compiled by experts to summarise research effort and AR status throughout Europe. The analyses provide heuristic prevalence estimates based on the best available data, which serve as a baseline for future AR research, guided by updated statistical guidelines [26]. This analytical approach also helps identify research limitations and opportunities for improvement. Despite available evidence of widespread AR in Europe, this study showed that harmonised surveys estimating its extent are scarce, lack standardisation, and are spatially biased at a country level. However, as the knowledge base on the AR status in livestock in Europe improves, this will be a stimulus for studies in additional countries, and for using more rigorous sampling strategies.

### Current status of anthelmintic resistance in Europe and reporting bias

Anthelmintic resistance in GIN was reported throughout Europe and was detected wherever studies were conducted (with some notable exceptions, e.g. Italy). Prevalence of AR in GIN against BZ, LEV, ML and MOX ranged between 0% and 48% when averaged over the entire database, but confidence intervals were wide and there was a high level of heterogeneity between studies. There was also considerable variation within and between regions depending on host species and anthelmintic class. Ten studies were largely in agreement that AR against MPTL in sheep is currently rare in Europe, although these studies were focussed on only six countries. AR in *F. hepatica* was reported in only five countries, and there were insufficient data to draw conclusions on AR in GIN against CLOS.

Although consultation with regional experts to compile unpublished datasets (almost a quarter of all publications), and implementing looser inclusion criteria, allowed the inclusion of data from more countries than in previous reviews [42], publications were still biased towards Western European countries. This appeared to be somewhat needs-led and justified by the estimated economic impact of helminth infections in these countries [9]. Nevertheless, spatial sampling bias, e.g. due to research expenditure, convenience sampling (e.g. close to research institutions or close to highways) or cultural differences (e.g. regional variability in response rates), can limit the analysis and interpretation of AR data. Furthermore, with some notable exceptions (e.g. studies of AR in cattle in Italy), there was a bias towards publishing positive findings: this is evident in the fact that the spatial country-level distribution of AR in Europe (https://www.parasite-journal.org/10.1051/parasite/2020062">Fig. S4) is reflected in the spatial distribution of publications per country (Fig. 2). Failing to account for these biases could result in incorrect or misleading inferences [7]. As a result, the current data are insufficient to conduct advanced geostatistical analyses.

### Research effort and priorities for future investment

The relationship between research effort and observed AR was investigated semi-quantitatively using composite indices of level of AR across anthelmintic groups and research effort or confidence, per country. Level of AR included the number of anthelmintic groups to which AR was reported and observed prevalence of resistance to each (i.e. proportion of farms with AR). Research effort, indicative of relative confidence in the levels of AR, comprised the number of studies in the database, the total number of farms investigated, and the number of anthelmintic groups tested. The positive relationship between level of AR and research effort in the better-studied systems (especially GIN in sheep) is to be expected for two main reasons. Firstly, as more studies are conducted and more anthelmintic groups tested, the chance of finding AR increases. Secondly, a perceived problem with anthelmintic efficacy is often the stimulus for investigations, and finding AR will provide justification for further and larger studies, fuelling a cycle of perceived and observed AR. To some extent, therefore, differences between countries in both observed level of treatment failure and research effort represent different positions on a trajectory of learning about existing AR problems, as well as between-country heterogeneity in true levels of AR. Nevertheless, the fact that low research effort is invariably associated with low apparent levels of treatment failure should stimulate further studies, especially in those countries positioned towards the lower left in Figure 4.

Overall confidence in AR status in GIN in cattle, and in fluke in both sheep and cattle, was very low, and further studies should also be prioritised to address these knowledge gaps in all countries. In sheep and goats, high confidence can be placed in high levels of AR in several countries (upper right regions in Fig. 4). The aim of further studies in these countries should be not so much to demonstrate a problem with anthelmintic efficacy, as to focus on solutions for farms facing resistance to multiple anthelmintic groups, including how newer groups (such as the aminoacetonitrile derivatives and spiroindole anthelmintics) should be used strategically, alongside the existing anthelmintic classes, to maintain the active life of both for as long as possible and allow for implementation of alternative approaches. Elsewhere, more reliable estimates of the prevalence of AR would be beneficial.

### Increase in prevalence of resistance across anthelmintic groups

Robust temporal analyses were hindered by small sample sizes (number of studies) and limited time range, as well as potential bias in farm selection. However, with the notable exception of LEV, for which some surveys gave inaccurate results before the mid-90s [19], successive studies following initial reports of AR invariably prove its existence, rather than identify such reports as exceptions to the overall situation, and this tendency suggests increasing AR over time. A trend of gradually increasing BZ resistance was evident both in aggregate (Fig. 5) and within more data-rich countries (Fig. 6). The later introduction of ML, especially MOX, and consequently more recent reports of AR in these groups make temporal analyses difficult, but a trend of increasing prevalence is also evident, at a faster rate than for BZ. This confirms a tendency towards shortening time lags between introduction of new anthelmintics to the market and appearance of resistance to them [26], which might indicate shared mechanisms of AR between some anthelmintic groups, differences in the nature of inheritance of resistance genes (e.g. dominant or recessive) or differences in initial abundance of resistance genes within the populations at release of product. Alongside research efforts to identify the scale of the problem, therefore, alternative strategies for sustainable parasite management, including targeted anthelmintic use, are essential and require significant new research [10, 37, 52].

Increasing AR is less obvious after 2009 for LEV, although sample sizes are low and there is no robust evidence of a decrease in prevalence. Studies in New Zealand have suggested that a change to less intensive treatment regimens might be successful in slowing or even reversing anthelmintic resistance in GIN populations [31]. The relatively low reported prevalence of AR in Italy despite high research effort also indicates that maintenance of anthelmintic efficacy is possible, and potentially attributable to aspects of farm management such as restraint in treatment of lactating ewes [41], although reports are biased to the south of the country where such factors pertain. Nevertheless, in countries for which longitudinal data were available, there was a clear trend towards increasing prevalence of AR. Average prevalence over the entire period studied, as shown in Figure 3, is therefore likely to underestimate the current scale of the problem. For example, in the UK the prevalence of resistance to BZ in sheep is reported as close to 50% over the whole period, but 82% in studies since 2010. Current high levels of resistance can therefore be concealed by a longer history of investigations while AR was less common. Conversely, high apparent prevalence could be generated by a few recent reports, which focus on farms where problems are suspected: thus, for BZ in sheep, AR prevalence of 100% in Poland and Romania results from a small number of single-farm studies.

The analyses of AR revealed several sources of bias, which can confound interpretation and impede solutions-oriented research. Thus, bias makes it very difficult to identify risk factors for rapid development of AR, and to determine whether differences in observed levels of treatment failure between countries, and between farms, can be used to guide future strategies. While removal of bias in studies of AR is difficult and perhaps impossible, greater efforts to reduce or account for it could support more refined analyses and better understanding of regional patterns and risk factors on which to base advice to farmers and policy makers.

### SWOT analysis

SWOT analysis also identified potential biases, and within-country variation that could affect current and future AR research. Although data protection legislation (especially EU Regulation 2016/679: General Data Protection Regulation; applicable from 2018) may limit the resolution of location data that can be published, there is now an opportunity to improve reporting of AR research to facilitate future geostatistical analyses. Future studies should strive to publish the location of farms in a way that protects farmer anonymity e.g. publishing latitudes and longitudes to one decimal place would give an indicative spatial accuracy of ±10 km at the equator.

During past decades, European research and investment tended to focus on resistance to antibiotics, but AR research effort accelerated after the turn of the millennium, coinciding with investment by the European Commission in several AR research consortia: PARASOL (€3.9M; https://cordis.europa.eu/project/id/22851) GLOWORM (€3M; https://cordis.europa.eu/project/id/288975/reporting), DELIVER (€3.6M; https://cordis.europa.eu/project/id/23025) and PARAGONE (€9M; https://cordis.europa.eu/project/id/635408). As a result, AR is now considered part of the broader antimicrobial resistance problem and stakeholders have recently recognised the emerging risk and agreed that it is now time to take concrete actions to ensure the responsible use of all veterinary medicines, including anthelmintics in livestock species [14].

### The need for standard methods for resistance detection and study design

Novel and harmonised protocols for the detection of AR in ruminants to include improved statistics and modified parasite enumeration methods that reduce measurement error [26, 53] are likely to improve the accuracy of AR diagnosis in future. This presents an opportunity for parallel attempts to reduce sampling error by introducing more robust randomised sampling protocols (e.g. [33]) and larger sample sizes. Greater accuracy in measuring AR would allow regional prevalence estimates to be refined and more advanced meta-analyses to be conducted to disentangle heterogeneity between studies arising from measurement error, sampling error, and true regional differences. The inclusion criteria for the current analysis were intentionally broad and a high level of trust was placed in the authors’ classification of resistance, especially where methodological detail was limited (e.g. in unpublished abstracts and reports); future studies would benefit from standardised and scalable methods for classification of AR, especially in liver fluke. Improved technologies for species identification, such as nemabiome [2] could also underpin more species-specific information on AR among GIN, and attenuate bias in AR estimates on mixed GIN populations.

Confidence in AR estimates is limited by the number of studies undertaken in each country, the number of farms included in each, and study design. The way in which farms were selected is also important. Preferential and opportunistic sampling is common in veterinary parasitology [6, 7], particularly where a phenotype or genotype is rare. Therefore, bias is to be expected, at least in the early stages of resistance to any active compound, as research effort will often focus on investigating farms reporting reduced efficacy of anthelmintics. Although fit for the original purpose of the study, this introduces difficulties for additional analysis and interpretation of findings, and comparison of results between regions. AR research generally lacks studies with large samples of randomly selected farms: the studies collated here most often sampled single farms close to universities or research institutes, and the median sample size for AR studies on GIN was only five farms. Even for larger, later studies that attempt to gain more comprehensive information, it is often impossible or impractical to select farms truly randomly, as logistics and farmer compliance are important limitations.

Non-random sampling, for example, due to researchers selecting farms with suspected AR, or farmers self-selecting to participate (possibly because they suspect they have AR on their farm) may inflate estimated prevalence. This is supported by a survey of both randomly selected and non-randomly selected sheep flocks in Norway [12] where the prevalence of BZ resistance in GIN on randomly selected farms in Rogaland (29%, *n* = 6) was lower than on non-randomly selected farms (80%, *n* = 10). Similarly, small sample sizes (e.g. due to resource limitations, since AR detection methods are time consuming for researchers and farmers) may inflate uncertainty. Study design includes the active compounds to be tested and whether or not to include an untreated control group, which are also subject to farmer consent: FECRT with all five licensed groups for sheep (including an avermectin and moxidectin) and a control group, with 15 animals in each, would require 105 animals, which is often unrealistic on commercial farms due to the time and cost of sampling; consequently, most studies test only a limited range of anthelmintics. The conditions for inclusion of animals in the test, such as pre-treatment Faecal Egg Count (FEC) varies between studies, as does the detection limit of the FEC method used, and the extent of species identification. The gold standard for assessing AR, the CET, requires slaughter of both control animals and treated animals, and is not suitable for monitoring on commercial farms. Each FECRT or *in vitro* method is open to error, e.g. FECs are an indirect measure of anthelmintic efficacy against adult helminths. Sample size within the existing database was not sufficiently high to compare AR prevalence across countries while taking account of variation in method and would be further undermined by concealed variation and bias.

### Slowing the future development of anthelmintic resistance

Frequent anthelmintic treatment is held as one of the major reasons for the genesis of resistant GIN populations, and a number of approaches are advocated to slow the development of AR [26]. Extensive research has been performed to establish strategies for targeted (group) and targeted selective treatments (single animal treatment), aiming at a reduction of the overall treatment frequency and amount of anthelmintic administered [8, 26]. There is a pressing need for improved, rapid diagnostics to facilitate routine monitoring and targeted treatment strategies. Nevertheless, two successful examples of monitoring programmes based on these strategies are reported from Switzerland and Italy. Experiences from monitoring programmes are well suited for dissemination to further countries.

In Switzerland, a programme was established in 1999 by the Small Ruminant Health Service to provide an annual service for sheep and goat farmers based on quantitative faecal examination. The programme, now including about 1400 farms, is partially subsidized by the government, covering about one third of the costs. Pooled samples are analysed from different groups of animals and, based on the results, veterinary advice is given with respect to anthelmintic treatment and further sampling. An evaluation performed five years after establishment of the program revealed that, on 73% of the farms, GIN were controlled successfully, with a mean annual treatment frequency of 2.0 compared with 3.4 on farms that did not participate in the programme. Participation in faecal monitoring also strengthened collaboration with the veterinarian – according to the farmers, the support of the veterinarian was “good” or “very good” in 86% of cases (unpublished data).

In Italy, the Regional Centre for Monitoring Parasitic Infections (Centro Regionale per il Monitoraggio delle Parassitosi – CREMOPAR) has been established since 2000 by the Department of Agriculture and Livestock Production of the Campania region and is economically supported by the farmers’ associations of Campania and other Italian regions. The strategies for the management of livestock infections caused by GIN, liver flukes and other parasites are based on promoting: best practice for diagnosis, regular monitoring on-farm (at least three FEC per year), best practice for treatment, monitoring the efficacy of treatments through the FECRT, and tailored advice to farm veterinarians on sustainable parasite control. These recommendations and activities are now well integrated into routine farm management of large ruminants (cattle and water buffaloes) and small ruminants (sheep and goats). The programme includes over 1000 farms per year. A survey performed for the last decade revealed that, on 88% of the farms, GIN were controlled successfully, with a lower mean annual treatment frequency of 2.0 compared with 3.1 on farms that did not participate in the programme. Participation in faecal monitoring also strengthened collaboration with the veterinarian – according to the farmers the support of veterinarian was “very good” in 94% of cases.

Recent studies suggest that improving awareness of helminth control and AR may improve the uptake of sustainable parasite control practices [50]. Jack et al. [24] found that farmers’ perceptions of AR risk (perceiving AR as being a problem in their region and a threat to their farming business) positively affected uptake of sustainable parasite control practices on Scottish sheep farms. In cattle farmers, Vande Velde et al. [51] found that a positive attitude towards anthelmintics was a barrier to the uptake of sustainable parasite control practices and that risk perception of anthelmintic resistance had no effect on the adoption intention of diagnostic methods, indicating that farmers did not yet consider AR a problem. Therefore, providing farmers and prescribers with access to information on the potential risk of AR in their region, such as the data presented here, may improve the uptake of sustainable control practices. This is supported by a similar social science veterinary epidemiology study of UK horse owners, which suggested that increasing perceived knowledge of helminth control and AR could improve the uptake of sustainable control practices [44]. Given between and within-country differences in livestock management and in cultural norms, research to understand uptake of sustainable control practices in individual European countries would be of benefit.

### Conclusions

AR is present across the European continent and despite the limitations of the current body of AR research, the analyses presented here provide useful indicators of the potential scale and potential future trajectory of the problem of AR in Europe, and may help improve the uptake of sustainable control practices. As the presence of AR in GIN is now widespread globally, and reports of AR in *F. hepatica* are increasing, confirming AR presence on small numbers of farms may no longer be necessary. The introduction of more rigorous methods for detecting AR is an opportunity to shift focus to employing robust randomised sampling methods that would allow more detailed epidemiological investigations, including improved estimates of regional prevalence and risk factor analysis.

## Conflicts of interest

The authors have no conflicts of interest to declare.

## Author contribution statements

LR, ERM and HRV conceived the project. HRV and LR collated data. HRV, ERM and LR conducted analyses and drafted the manuscript. All authors developed the database by contributing data and participating in quality control, assisted with technical editing of the manuscript, and approved the final manuscript and supplementary data files.

## Supplementary Materials

The supplementary material of this article is available at https://www.parasite-journal.org/10.1051/parasite/2020062 *Figure S1*. Number of publications (peer-reviewed papers or theses) investigating AR in GIN in ruminants.
*Figure S2*. Estimated prevalence of resistance against the benzimidazoles (BZ), levamisole (LEV), avermectins (ML; macrocyclic lactones), and moxidectin (MOX) in gastrointestinal nematodes in goats.
*Figure S3*. Estimated prevalence of resistance against the benzimidazoles (BZ), levamisole (LEV), avermectins (ML; macrocyclic lactones), and moxidectin (MOX) in gastrointestinal nematodes in cattle.
*Figure S4*. Countries reporting at least one case of anthelmintic resistance in gastrointestinal nematodes infecting sheep, goats and cattle in Europe.
*Table S1.* Mean (SD in brackets) sample size weighted prevalence of anthelmintic resistance in sheep stratified by country and anthelmintic class.
*Table S2.* Mean (SD in brackets) sample size weighted prevalence of anthelmintic resistance in cattle stratified by country and anthelmintic class.
*Table S3.* Mean (SD in brackets) sample size weighted prevalence of anthelmintic resistance in goats stratified by country and anthelmintic class.
*Table S4*. Regression analysis results for prevalence of anthelmintic failure over time.
*Table S4a*. Benzimidazole efficacy in sheep in Switzerland.
*Table S4b*. Benzimidazole efficacy in sheep in the UK.
*Table S4c*. Ivermectin efficacy in sheep in the UK.

## Notes

**Cite this article as**: Rose Vineer H, Morgan ER, Hertzberg H, Bartley DJ, Bosco A, Charlier J, Chartier C, Claerebout E, de Waal T, Hendrickx G, Hinney B, Höglund J, Jeek J, Kašný M, Keane OM, Martínez-Valladares M, Mateus TL, McIntyre J, Mickiewicz M, Munoz AM, Phythian CJ, Ploeger HW, Rataj AV, Skuce PJ, Simin S, Sotiraki S, Spinu M, Stuen S, Thamsborg SM, Vadlejch J, Varady M, von Samson-Himmelstjerna G & Rinaldi L. 2020. Increasing importance of anthelmintic resistance in European livestock: creation and meta-analysis of an open database. Parasite **27**, 69.
