# Supplementary material for: Increasing importance of anthelmintic resistance in European livestock: creation and meta-analysis of an open database
Source: Parasite. 2020 Dec 4;27:69. doi: 10.1051/parasite/2020062 (PMC7718593; doi:10.1051/parasite/2020062)
Supplement: Supplementary file 1 — Figure S1. Number of publications (peer-reviewed papers or theses) investigating AR in GIN in ruminants. Figure S2. Estimated prevalence of resistance against the benzimidazoles (BZ), levamisole (LEV), avermectins (ML; macrocyclic lactones), and moxidectin (MOX) in gastrointestinal nematodes in goats. Figure S3. Estimated prevalence of resistance against the benzimidazoles (BZ), levamisole (LEV), avermectins (ML; macrocyclic lactones), and moxidectin (MOX) in gastrointestinal nematodes in cattle. Figure S4. Countries reporting at least one case of anthelmintic resistance in gastrointestinal nematodes infecting sheep, goats and cattle in Europe. Table S1. Mean (SD in brackets) sample size weighted prevalence of anthelmintic resistance in sheep stratified by country and anthelmintic class. Table S2. Mean (SD in brackets) sample size weighted prevalence of anthelmintic resistance in cattle stratified by country and anthelmintic class. Table S3. Mean (SD in brackets) sample size weighted prevalence of anthelmintic resistance in goats stratified by country and anthelmintic class. Table S4. Regression analysis results for prevalence of anthelmintic failure over time. Table S4a. Benzimidazole efficacy in sheep in Switzerland. Table S4b. Benzimidazole efficacy in sheep in the UK. Table S4c. Ivermectin efficacy in sheep in the UK. [file parasite-27-69-s1.pdf]

Supplementary material:

Figure S1. Number of publications (peer-reviewed papers or theses) investigating AR in GIN in ruminants.

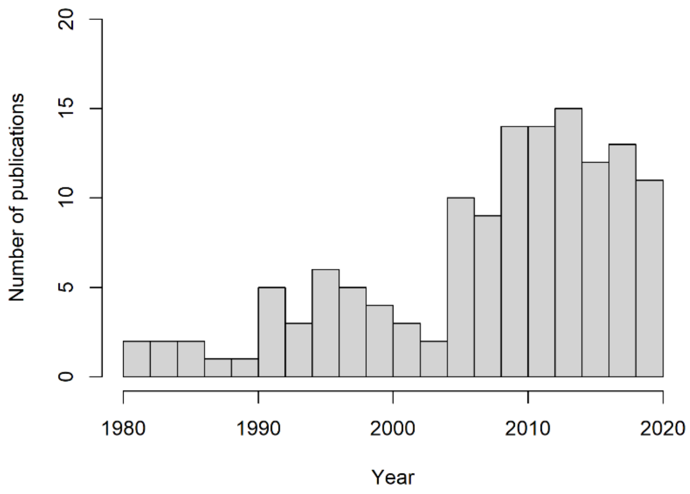

Figure S2. Estimated prevalence of resistance against the benzimidazoles (BZ), levamisole (LEV), avermectins (ML; macrocyclic lactones), and moxidectin (MOX) in gastrointestinal nematodes in goats. Points and whiskers represent the weighted prevalence estimate and standard deviation, respectively. The weighted prevalence and standard deviation across all studies are represented by the dashed black and grey vertical ablines, respectively. Note that points without whiskers represent single studies, for which standard deviations could not be estimated (i.e. they do not represent points for which the prevalence is known with a high level of confidence).

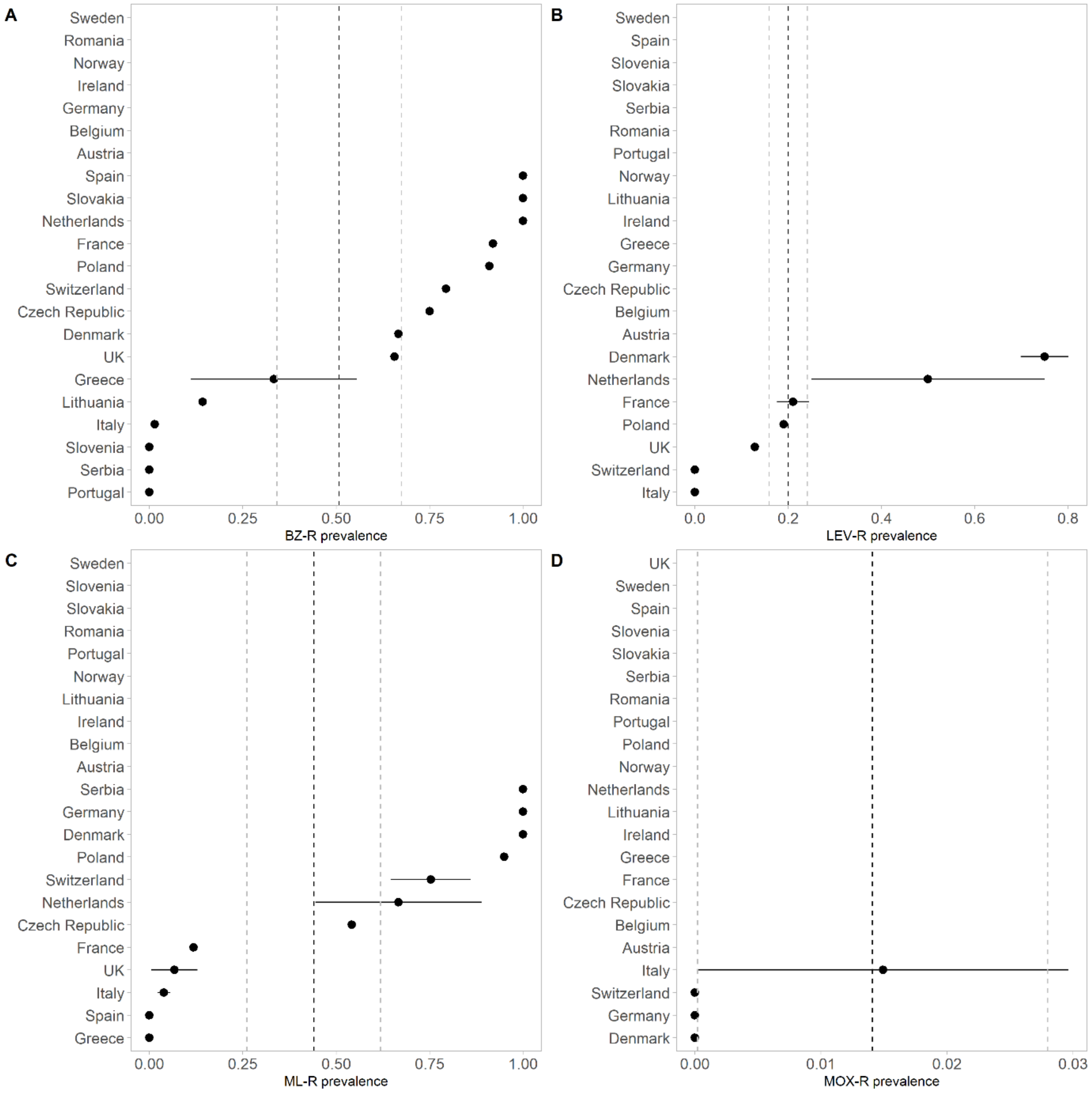

Figure S3. Estimated prevalence of resistance against the benzimidazoles (BZ), levamisole (LEV), avermectins (ML; macrocyclic lactones), and moxidectin (MOX) in gastrointestinal nematodes in cattle. Points and whiskers represent the weighted prevalence estimate and standard deviation, respectively. The weighted prevalence and standard deviation across all studies are represented by the dashed black and grey vertical ablines, respectively. Note that points without whiskers represent single studies, for which standard deviations could not be estimated (i.e. they do not represent points for which the prevalence is known with a high level of confidence).

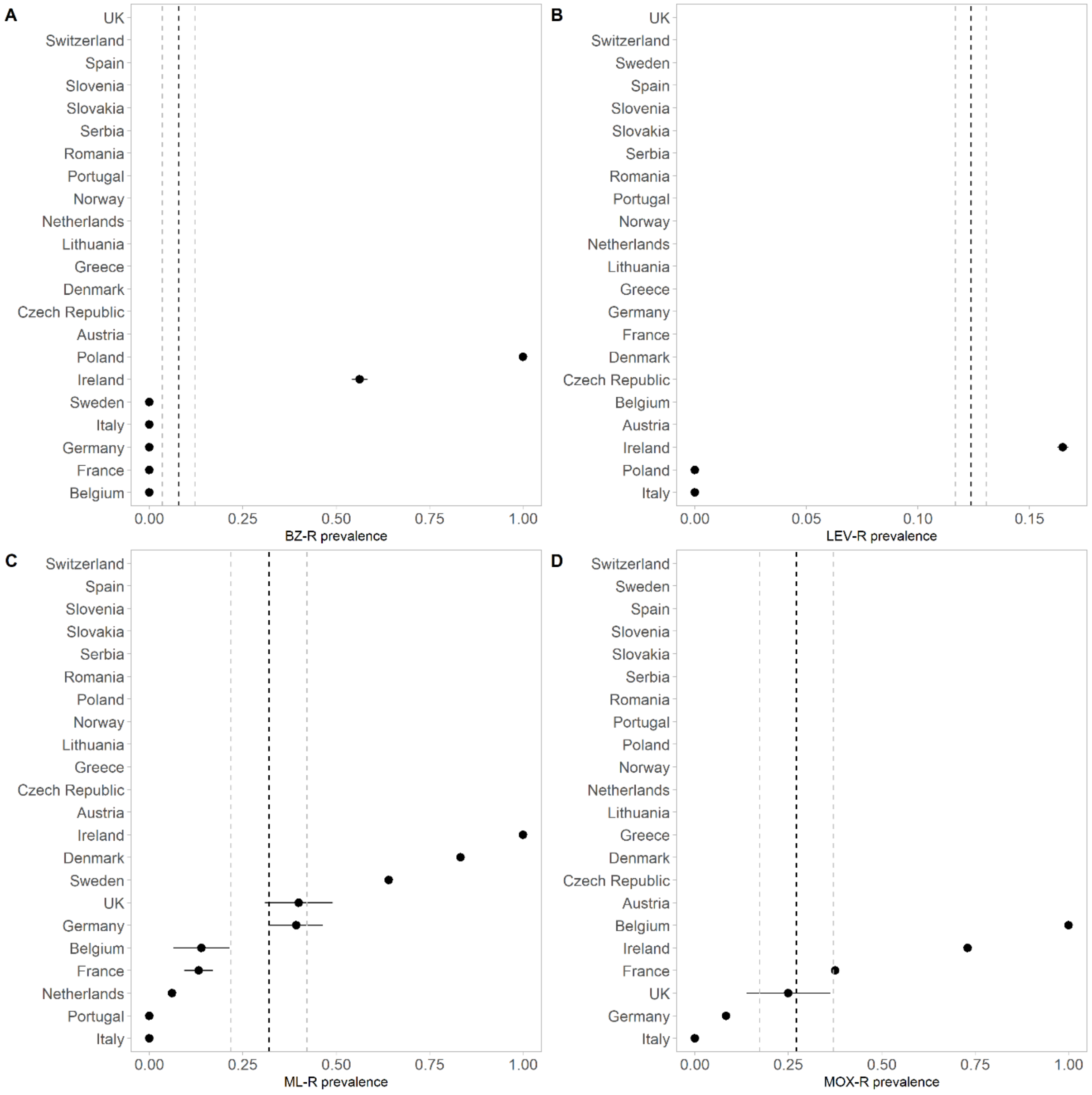

Figure S4. Countries reporting at least one case of anthelmintic resistance in gastrointestinal nematodes infecting sheep, goats and cattle in Europe, against any anthelmintic class (green). Countries with no data are shown in grey. The “no data” category includes countries with no published reports of AR, therefore the presence of AR n these countries cannot be ruled out. Regional variation within countries is not shown due to insufficient data resolution.

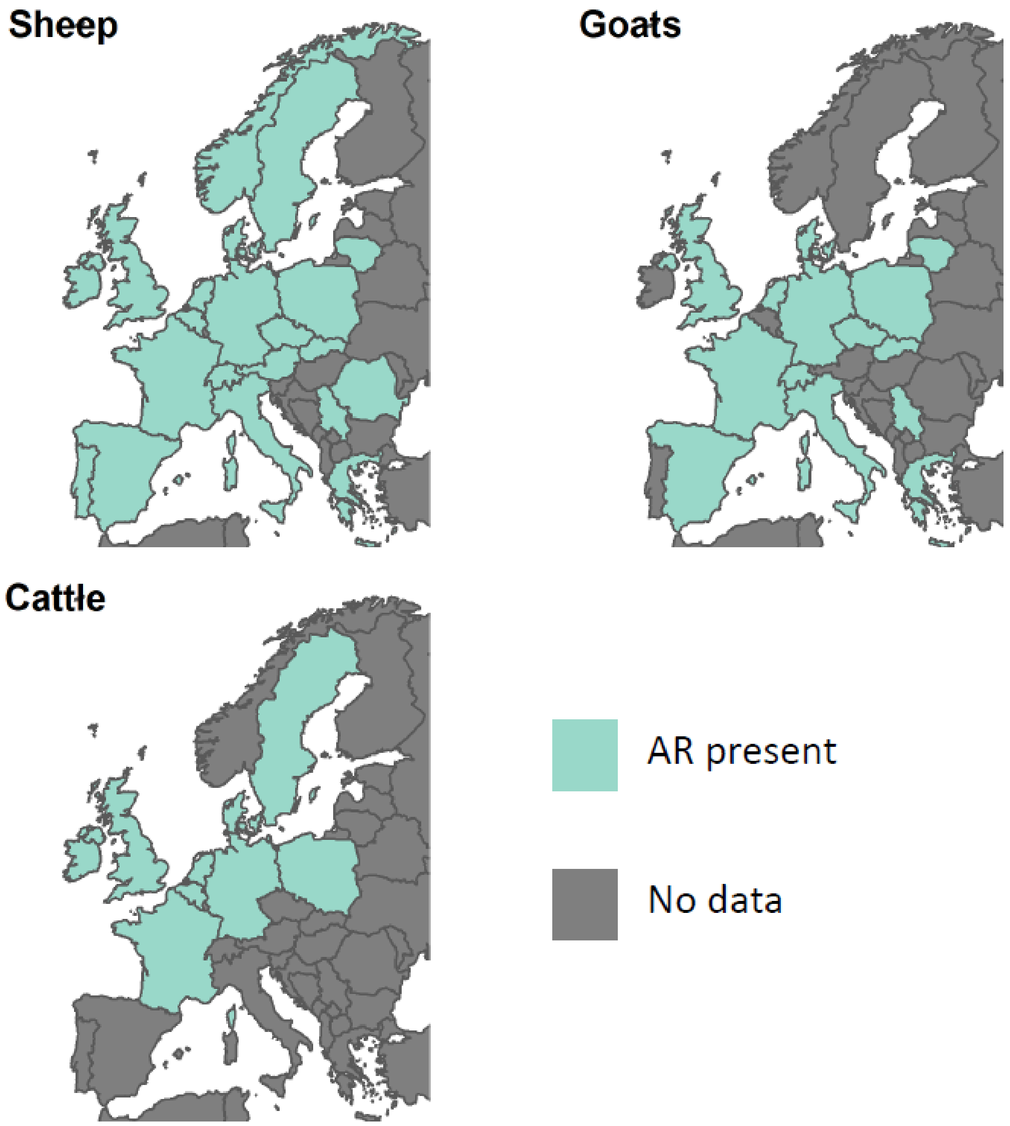

Table S1. *Mean (SD in brackets) sample size weighted prevalence of anthelmintic resistance in sheep stratified by country and anthelmintic class. BZ = benzimidazole, LEV = levamisole, ML = avermectins (macrocyclic lactones), MOX = moxidectin, MPTL = monepantel, CLOS = closantel, - = no data available. Number of studies (n; number of publications in brackets) indicates the number of groups tested, which may be >1 per publication. For example, some publications provide results for multiple regions.*

Country	*BZ*	*n*	*LEV*	*n*	*ML*	*n*	*MOX*	*n*	*MPTL*	*n*	*CLOS*	*n*
Austria	0.85 (0.02)	3 (3)	-	0 (0)	-	0 (0)	0.12 (0.01)	2 (2)	-	0 (0)	-	0 (0)
Belgium	1 (0)	3 (2)	-	0 (0)	0.71 (0.03)	2 (1)	0.12 (0)	2 (2)	1 (0)	1 (1)	-	0 (0)
Czech Republic	0.4 (0)	1 (1)	-	0 (0)	0.1 (0)	1 (1)	-	0 (0)	-	0 (0)	-	0 (0)
Denmark	0.3 (0.02)	3 (3)	0.67 (0.05)	3 (3)	0.62 (0.02)	2 (2)	0 (0)	1 (1)	-	0 (0)	-	0 (0)
France	0.9 (0.01)	8 (7)	0.52 (0.02)	4 (4)	0.21 (0.06)	6 (6)	0.11 (0.02)	5 (5)	0 (0)	1 (1)	0 (0)	1 (1)
Germany	0.7 (0.01)	3 (3)	0 (0)	1 (1)	0 (0)	1 (1)	0.19 (0.01)	3 (3)	-	0 (0)	-	0 (0)
Greece	0.81 (0.1)	4 (3)	0.5 (0.11)	2 (1)	0.1 (0.02)	2 (1)	0.1 (0.02)	2 (1)	-	0 (0)	-	0 (0)
Ireland	0.7 (0)	4 (4)	0.48 (0)	3 (3)	0.5 (0)	1 (1)	0.16 (0)	2 (2)	-	0 (0)	-	0 (0)
Italy	0.11 (0.07)	14 (10)	0.3 (0.11)	3 (3)	0.18 (0.12)	10 (9)	0 (0)	3 (3)	0 (0)	2 (2)	-	0 (0)
Slovenia	0 (0)	1 (1)	-	0 (0)	-	0 (0)	-	0 (0)	-	0 (0)	-	0 (0)
Spain	0.13 (0)	7 (7)	0.47 (0.02)	3 (3)	0.22 (0.02)	6 (5)	0.03 (0)	1 (1)	-	0 (0)	-	0 (0)
Sweden	0.05 (0)	3 (3)	0 (0)	1 (1)	0.06 (0.04)	4 (3)	0 (0)	1 (1)	-	0 (0)	-	0 (0)
Lithuania	0.73 (0.14)	3 (3)	-	0 (0)	0.41 (0.06)	2 (2)	-	0 (0)	-	0 (0)	-	0 (0)

Netherlands	0.62 (0.09)	9 (9)	0.07 (0)	5 (5)	0.34 (0.14)	8 (7)	0.34 (0.03)	7 (7)	0.11 (0.03)	3 (3)	0.27 (0)	1 (1)
Norway	0.29 (0.09)	17 (5)	-	0 (0)	0.01 (0.01)	14 (3)	-	0 (0)	-	0 (0)	-	0 (0)
Poland	1 (0)	1 (1)	0 (0)	1 (1)	-	0 (0)	-	0 (0)	-	0 (0)	-	0 (0)
Portugal	1 (0)	2 (2)	-	0 (0)	0.2 (0.16)	2 (2)	-	0 (0)	-	0 (0)	-	0 (0)
Romania	1 (0)	1 (1)	-	0 (0)	-	0 (0)	-	0 (0)	-	0 (0)	-	0 (0)
Serbia	-	0 (0)	-	0 (0)	1 (0)	1 (1)	-	0 (0)	-	0 (0)	-	0 (0)
Slovakia	0.2 (0.11)	5 (5)	-	0 (0)	0.23 (0)	1 (1)	-	0 (0)	-	0 (0)	-	0 (0)
Switzerland	0.95 (0.01)	5 (5)	-	0 (0)	0.17 (0)	1 (1)	0.5 (0)	1 (1)	1 (0)	1 (1)	-	0 (0)
UK	0.53 (0.07)	26 (22)	0.23 (0.06)	17 (16)	0.47 (0.06)	17 (15)	0.37 (0.05)	6 (6)	0.5 (0.25)	2 (2)	-	0 (0)

Table S2. *Mean (SD in brackets) sample size weighted prevalence of anthelmintic resistance in cattle stratified by country and anthelmintic class. BZ = benzimidazole, LEV = levamisole, ML = avermectins (macrocyclic lactones), MOX = moxidectin, MPTL = monepantel, CLOS = closantel, - = no data available. Number of studies (n; number of publications in brackets) indicates the number of groups tested, which may be >1 per publication. For example, some publications provide results for multiple regions.*

Country	*BZ*	*n*	*LEV*	*n*	*ML*	*n*	*MOX*	*n*	*MPTL*	*n*	*CLOS*	*n*
Austria	-	0 (0)	-	0 (0)	-	0 (0)	-	0 (0)	-	0 (0)	-	0 (0)
Belgium	0 (0)	1 (1)	-	0 (0)	0.14 (0.08)	4 (4)	1 (0)	1 (1)	-	0 (0)	-	0 (0)
Czech Republic	-	0 (0)	-	0 (0)	-	0 (0)	-	0 (0)	-	0 (0)	-	0 (0)
Denmark	-	0 (0)	-	0 (0)	0.83 (0)	1 (1)	-	0 (0)	-	0 (0)	-	0 (0)
France	0 (0)	2 (2)	-	0 (0)	0.13 (0.04)	4 (4)	0.38 (0)	1 (1)	-	0 (0)	-	0 (0)
Germany	0 (0)	1 (1)	-	0 (0)	0.39 (0.07)	3 (3)	0.08 (0)	1 (1)	-	0 (0)	-	0 (0)
Greece	-	0 (0)	-	0 (0)	-	0 (0)	-	0 (0)	-	0 (0)	-	0 (0)
Ireland	0.56 (0.02)	2 (2)	0.16 (0)	2 (2)	1 (0)	2 (2)	0.73 (0)	1 (1)	-	0 (0)	-	0 (0)
Italy	0 (0)	7 (5)	0 (0)	1 (1)	0 (0)	6 (6)	0 (0)	3 (3)	0 (0)	1 (1)	-	0 (0)
Slovenia	-	0 (0)	-	0 (0)	-	0 (0)	-	0 (0)	-	0 (0)	-	0 (0)
Spain	-	0 (0)	-	0 (0)	-	0 (0)	-	0 (0)	-	0 (0)	-	0 (0)
Sweden	0 (0)	1 (1)	-	0 (0)	0.64 (0)	4 (4)	-	0 (0)	-	0 (0)	-	0 (0)
Lithuania	-	0 (0)	-	0 (0)	-	0 (0)	-	0 (0)	-	0 (0)	-	0 (0)

Netherlands	-	0 (0)	-	0 (0)	0.06 (0)	1 (1)	-	0 (0)	-	0 (0)	-	0 (0)
Norway	-	0 (0)	-	0 (0)	-	0 (0)	-	0 (0)	-	0 (0)	-	0 (0)
Poland	1 (0)	1 (1)	0 (0)	1 (1)	-	0 (0)	-	0 (0)	-	0 (0)	-	0 (0)
Portugal	-	0 (0)	-	0 (0)	0 (0)	1 (1)	-	0 (0)	-	0 (0)	-	0 (0)
Romania	-	0 (0)	-	0 (0)	-	0 (0)	-	0 (0)	-	0 (0)	-	0 (0)
Serbia	-	0 (0)	-	0 (0)	-	0 (0)	-	0 (0)	-	0 (0)	-	0 (0)
Slovakia	-	0 (0)	-	0 (0)	-	0 (0)	-	0 (0)	-	0 (0)	-	0 (0)
Switzerland	-	0 (0)	-	0 (0)	-	0 (0)	-	0 (0)	-	0 (0)	-	0 (0)
UK	-	0 (0)	-	0 (0)	0.4 (0.09)	5 (5)	0.25 (0.11)	2 (2)	-	0 (0)	-	0 (0)

Table S3. *Mean (SD in brackets) sample size weighted prevalence of anthelmintic resistance in goats stratified by country and anthelmintic class. BZ = benzimidazole, LEV = levamisole, ML = avermectins (macrocyclic lactones), MOX = moxidectin, MPTL = monepantel, CLOS = closantel, - = no data available. Number of studies (n; number of publications in brackets) indicates the number of groups tested, which may be >1 per publication. For example, some publications provide results for multiple regions.*

Country	*BZ*	*n*	*LEV*	*n*	*ML*	*n*	*MOX*	*n*	*MPTL*	*n*	*CLOS*	*n*
Austria	-	0 (0)	-	0 (0)	-	0 (0)	-	0 (0)	-	0 (0)	-	0 (0)
Belgium	-	0 (0)	-	0 (0)	-	0 (0)	-	0 (0)	-	0 (0)	-	0 (0)
Czech Republic	0.75 (0)	1 (1)	-	0 (0)	0.54 (0)	1 (1)	-	0 (0)	-	0 (0)	-	0 (0)
Denmark	0.67 (0.01)	2 (2)	0.75 (0.05)	2 (2)	1 (0)	2 (2)	0 (0)	1 (1)	-	0 (0)	-	0 (0)
France	0.92 (0)	5 (5)	0.21 (0.03)	2 (2)	0.12 (0.01)	2 (2)	-	0 (0)	-	0 (0)	-	0 (0)
Germany	-	0 (0)	-	0 (0)	1 (0)	2 (2)	0 (0)	1 (1)	-	0 (0)	-	0 (0)
Greece	0.33 (0.22)	2 (2)	-	0 (0)	0 (0)	1 (1)	-	0 (0)	-	0 (0)	-	0 (0)
Ireland	-	0 (0)	-	0 (0)	-	0 (0)	-	0 (0)	-	0 (0)	-	0 (0)
Italy	0.01 (0.01)	6 (4)	0 (0)	2 (2)	0.04 (0.02)	5 (4)	0.01 (0.01)	3 (3)	0 (0)	2 (2)	-	0 (0)
Slovenia	0 (0)	1 (1)	-	0 (0)	-	0 (0)	-	0 (0)	-	0 (0)	-	0 (0)
Spain	1 (0)	1 (1)	-	0 (0)	0 (0)	1 (1)	-	0 (0)	-	0 (0)	-	0 (0)
Sweden	-	0 (0)	-	0 (0)	-	0 (0)	-	0 (0)	-	0 (0)	-	0 (0)
Lithuania	0.14 (0)	1 (1)	-	0 (0)	-	0 (0)	-	0 (0)	-	0 (0)	-	0 (0)

Netherlands	1 (0)	2 (2)	0.5 (0.25)	2 (2)	0.67 (0.22)	3 (2)	-	0 (0)	-	0 (0)	-	0 (0)
Norway	-	0 (0)	-	0 (0)	-	0 (0)	-	0 (0)	-	0 (0)	-	0 (0)
Poland	0.91 (0)	1 (1)	0.19 (0)	1 (1)	0.95 (0)	1 (1)	-	0 (0)	-	0 (0)	-	0 (0)
Portugal	0 (0)	1 (1)	-	0 (0)	-	0 (0)	-	0 (0)	-	0 (0)	-	0 (0)
Romania	-	0 (0)	-	0 (0)	-	0 (0)	-	0 (0)	-	0 (0)	-	0 (0)
Serbia	0 (0)	1 (1)	-	0 (0)	1 (0)	2 (2)	-	0 (0)	-	0 (0)	-	0 (0)
Slovakia	1 (0)	1 (1)	-	0 (0)	-	0 (0)	-	0 (0)	-	0 (0)	-	0 (0)
Switzerland	0.79 (0.01)	4 (4)	0 (0)	1 (1)	0.75 (0.11)	5 (5)	0 (0)	2 (2)	-	0 (0)	-	0 (0)
UK	0.66 (0)	2 (2)	0.13 (0)	1 (1)	0.07 (0.06)	2 (2)	-	0 (0)	-	0 (0)	-	0 (0)

Table S4. Regression analysis results for prevalence of anthelmintic failure over time.

This analysis corresponds to the results presented in Figure 6 in the main manuscript, and concerns the prevalence of anthelmintic failure (i.e., the proportion of farms testing at < 95% faecal egg count reduction per study) over time (year) in the three country-anthelmintic class combinations with sufficient data to permit analysis. ANOVA = analysis of variance; SS = sum of squares; MS = mean square; c = regression y-intercept, m = slope; CI = confidence interval.

Table S4a. Benzimidazole efficacy in sheep in Switzerland. *Adjusted R^2 = 0.86, regression coefficients (95% CI): c = -43.8 (-86.6 to -1.0) m = 0.022 (0.0009-0.0437). ANOVA:*

	df	SS	MS	F	p
Regression	1	0.3093	0.3093	20.011	0.0465
Residual	2	0.0309	0.0155		
Total	3	0.3402			

Table S4b. Benzimidazole efficacy in sheep in the UK. *Adjusted R^2 = 0.40, regression coefficients (95% CI): c = -39.1 (-61.1 to -17.1) m = 0.020 (0.0089-0.0308). ANOVA:*

	df	SS	MS	F	p
Regression	1	0.8177	0.8177	14.305	0.0013
Residual	19	1.0861	0.0572		
Total	20	1.9039			

Table S4c. Ivermectin efficacy in sheep in the UK. *Adjusted R^2 = 0.63, regression coefficients (95% CI): c = -66.3 (-101.5 to -31.1) m = 0.033 (0.0157-0.0508). ANOVA:*

	df	SS	MS	F	p
Regression	1	0.6433	0.6433	18.390	0.0020
Residual	9	0.3148	0.0350		
Total	10	0.9581			
